# Medicine in the Light of Evolution

**DOI:** 10.3390/genes10010003

**Published:** 2018-12-21

**Authors:** Olga Dolgova, Oscar Lao

**Affiliations:** Population Genomics Group, Centre Nacional d’Anàlisi Genòmica, Centre de Regulació Genòmica (CRG-CNAG), Parc Científic de Barcelona, Baldiri Reixac 4, 08028 Barcelona, Catalonia, Spain

Evolutionary medicine applies the principles of evolutionary biology to understand *why* we get sick rather than *how*, and it has undergone an exponential growth since the early 1990s [1]. Evolutionary theory considers that every trait is a result of selective constraints and phenotypical trade-offs. The final goal of natural selection is shaping organisms for maximum reproductive success rather than for supporting their health. This explains why mutations that increase reproduction spread, even if they decrease health and longevity. Within this evolutionary context, the reasons for the existence of a given pathology are of diverse nature. A subtle and recent shift of the environment, including lifestyle and habits, can expose humans to new infectious pathogens and produce unforeseen pathologies in the original environment. Humans have managed to spread all over the world after the Out of Africa event in an extremely short period from an evolutionary point of view. Conquering such a wide range of different environments has been possible by biological adaptation including interbreeding with archaic hominins and mostly by the continuous discovery of new technological advances and cultural adaptations. Of these, the discovery of agriculture ~10 thousand years ago in different regions of the world was of particular relevance, both in terms of human demography, behavior and environmental changes. Nevertheless, cultural adaptation has not stopped, but accelerated during the industrial revolution -less than 200 years ago- and even more with the digital revolution less than 50 years ago. All these cultural changes are meant to help humankind by changing the environment towards facilitating/reducing physical tasks, minimizing the energetic costs to get high caloric food and reducing parasitic and infectious diseases exposure by increasing sanitation and antibiotics, among others. Because of all these cultural changes, current environment where humans live is far from being the one where we biologically evolved [2].

Furthermore, infectious pathogens evolve ways to deal with antibiotics and the protective defenses of the human body. This coevolutionary arms race shapes virulence levels to maximize the rate of pathogen spread. Vulnerability also arises from physical constraints resulting from the tinkerer nature of selection and the random nature of mutational events, for example, the blind spot in the eyes of vertebrates or inevitability of DNA replication errors. Additionally, many symptoms are not diseases but protective responses shaped by natural selection, such as pain, fever, cough, and anxiety. The cost of many bodily defenses is low compared with the cost of not expressing a defense when it is needed, so the normal mechanisms shaped by natural selection give rise to many false alarms and apparently excessive responses [3].

Taking these multiple disease origins into account, evolutionary thinking helps to understand and manage most health challenges in the modern world, including emerging infectious disease, evolution of antimicrobial resistance, aging, reproductive health, increasing prevalence of autoimmune diseases and immune function, obesity epidemics, threats to food safety and diet, neurodegenerative diseases, behavioral disorders and mental health, cancer, microbiomes, veterinary medicine, inflammation, among others. While some evolutionary research programs implement evolutionary approaches to more effectively prevent or treat diseases, others aim to account for the vulnerability of humans to illnesses. The principles of population genomics and human evolutionary history attain special importance in the field of personalized medicine, which involves genetic tests and targeted interventions used for risk predictions, treatment decisions, or prenatal screening focusing on either the individual genetic background or the somatic genetic variation acquired, for example, during tumor transformation. Furthermore, the scope of personalized medicine currently begins to take into account not only differences in genetic information, but also in environments and lifestyle [4], making evolutionary approaches especially relevant. Addressing this wide range of questions, evolutionary medicine proves to be a powerful and broad lens for medical advancement, which integrates knowledge and approaches of evolution, ecology, biological anthropology, population genomics, and global health.

The growing area of NGS technologies has allowed the investigation of how different pathogens influenced the evolution of humanity and particular components of the human genome. For example, we know that more than 4000 of the 25,000 total genes in the human genome interact with pathogens; furthermore, about one-third of protein adaptations since humans split from other great apes was driven by a response to infectious viruses [5], which modern humans had been facing along their evolutionary history and geographic expansion. It was also found that proteins that interact with viruses evolve under stronger purifying selection and tend to adapt at much higher rates compared to similar proteins that do not interact with viruses [5]. Furthermore, whole genome sequencing applied recently to ancient genomes discovered archaic fragments in modern human genomes. This evidence of interbreeding with archaic populations has brought to a new perspective our understanding of disease and disease resistance evolution. While there is evidence that most introgressed DNA segments were removed by purifying selection from the modern human genome, some stayed and quickly increased to high frequencies at the time of contact, suggesting a selective advantage. When modern humans interbred with Neanderthals, they infected each other with pathogens that came from their respective environments [6]. At the same time, they also passed along genetic adaptations to cope with some of those pathogens. Resistance to specific RNA viruses provided by Neanderthal sequences was likely a big part of the reason for their selective benefits [6]. Recent studies have also detected specific non-disease phenotypic consequences of archaic introgression. Examples are skin pigmentation and hair color, height, sleeping patterns, mood, and smoking status in present-day Europeans [7] and hemoglobin concentration and response to hypoxia in Tibetans [8,9], associated with signatures of positive selection as evidence of their benefit for modern human survival in new territories in the past as well as archaic contribution to the susceptibility to different pathologies in modern humans [10] supposedly due to the environmental mismatches.

In this Special Issue, the authors trace the evolutionary grounds of many disease and health genotypes and phenotypes using a variety of approaches. A couple of reviews deal with the new insights that the sequencing of ancient DNA (aDNA) is providing into the history of human pathologies. Dolgova and Lao [11] review the evolutionary and medical implications of archaic interbreeding in current human populations, elucidating new interpretations on the potential impact of introgression on fitness and the role of natural selection within the context of health and disease.

Pimenoff et al. [12] explain the role of aDNA in the understanding of ancient viral–host parallel evolutionary histories in human sexually transmitted infections by examination of three groups of pathogens and providing insight into ancient human behavior and history. The exploration of the ancient viral genomic divergence and its coevolutionary patterns allows us to predict the potential environmentally induced responses in modern pathogens.

Understanding how drug resistance can evolve within naturally susceptible pathogenic species is key for developing novel, more effective treatment strategies. In their review, Ksiezopolska and Gabaldón [13] highlight the need of in-depth studies of drug resistance evolution in fungal species, much less investigated than antibiotic resistance in bacteria, and summarize current knowledge on antifungal drug resistance in Candida pathogens, focusing on the possible evolutionary paths that may lead to the emergence and spread of resistant phenotypes.

Gu et al. [14] explore the patterns of recent positive selection acting on human alcohol dehydrogenase (ADH)1B × 48His allele, which had rapidly and independently increasing in frequency in two populations of East and Southwest Asia after their divergence, providing a beautiful example of convergent evolution probably as a response to the emergence of agriculture in both regions.

Cancer is a disease driven by somatic mutations that increases survival and proliferation of cell lineages as well as the evolution of genes associated with cancer risk in populations. Vicents and Posada [15] identified 40 cancer genes with a robust signal of positive selection across mammalian lineages among 430 genes associated with cancer. They provided evidence for fewer selective constraints and higher incidence of positive selection in cancer genes affecting germline and recessive mutations that predispose to cancer. This finding suggests a potential association between relaxed selection, positive selection, and risk of hereditary cancer.

Riley et al. [16] reject consistently the possibility of associations between mitochondrial DNA (mtDNA) haplogroups and breast cancer risk reported by other studies based on small sample sizes and inadequate statistical tests lacking population stratification. After rigorous examination of possible associations between of mtDNA haplogroups from extended Avon Longitudinal Study of Parents and Children (ALSPAC) birth cohort of European origin and over 40 independent breast cancer risk factor-related variables, they conclude that mitochondrial DNA haplogroups are unlikely to underlie susceptibility to breast cancer that occurs via the risk factors examined in this study of a population of European ancestry.

Obesity constitutes a case example of a modern trait shaped by contemporary environment. The extent to which gene-by-environment (G × E) interactions accentuate obesity risk in individuals following obesogenic lifestyles is still uncertain. There is accumulating evidence at the genome-wide level for a role of polygenic risk-by-environment interactions. Nagpal et al. [17] confirm that the genomic background plays a major role in shaping the expressivity of alleles that increase body mass index (BMI), lending support for interactions between genes and environments being a key factor in the architecture of complex traits.

Evolutionary insights have certainly influenced medical research in the last decades interplaying with growing amounts of human genomic data. The benefit of such influence is that it helps investigators to trace and understand the roots of any disease along the human evolutionary history, uncover new clues about ancient pathologies, and inform better ways to monitor for and treat future epidemics.

Overall, these studies show that Dobzhansky’s renowned dictum “Nothing in biology makes sense except in the light of evolution” is also extensible to medical research. An evolutionary perspective of medicine works as a comprehensive scaffolding for organizing medical principles and concepts (as it does in biology in general) that otherwise would remain unconnected [18].
